# Quantifying the climate benefits of a virtual versus an in-person format for an international conference

**DOI:** 10.1186/s12940-022-00883-7

**Published:** 2022-07-19

**Authors:** Jacqueline R. Lewy, Casey D. Patnode, Philip J. Landrigan, Joseph C. Kolars, Brent C. Williams

**Affiliations:** 1 University of Michigan Medical School, 6312D Med Sci I, SPC 5624, 1301 Catherine St., Ann Arbor, MI 48109-5624 USA; 2 Boston College, 140 Commonwealth Avenue, Chestnut Hill, MA 02467 USA; 3 Centre Scientifique de Monaco, 8 Quai Antoine 1er, 98000 Monaco City, Monaco

**Keywords:** Climate change, Planetary health, Environmental health, Global health, Public health, Academic conferences, Virtual conferences, Accessibility

## Abstract

**Background:**

Academic institutions across the globe routinely sponsor large conferences. During the COVID-19 pandemic, many conferences have used all- or partially virtual formats. The conversion of the 2021 Consortium of Universities for Global Health (CUGH) conference, originally planned in-person for Houston, TX USA to an all-virtual format provided an opportunity to quantify the climate-related impacts of in-person versus virtual conferences.

**Methods:**

From the 2021 CUGH conference registration data, we determined each registrant’s distance from Houston. Using widely available, open-source formulas, we calculated the carbon footprint of each registrant’s round-trip drive or flight had they traveled to Houston. We assumed that registrants traveling more than 300 miles would have flown, with the remainder traveling by automobile.

**Results:**

Of 1909 registrants, 1447 would have traveled less than 4000 miles, and 389 would have traveled more than 10,000 miles round trip. Total travel-related carbon emissions were estimated at 2436 metric tons of CO_2_, equivalent to the conservation of 2994 acres of forest for a year.

**Conclusions:**

Organizations can now readily quantify the climate cost of annual conferences. CUGH’s annual international conference, when held in-person, contributes significantly to carbon emissions. With its focus on promoting global health equity, CUGH may play a lead role in understanding the pros and cons for planetary health of in-person versus virtual conferences. CUGH and other organizations could routinely measure and publish the climate costs of their annual conferences.

## Background

Climate change is the greatest threat to global health facing the world in this century [1]. Prominent organizations such as *The Lancet*, *Nature*, and the WHO have sounded the alarm on the increasing devastation of planetary support systems, predicting that climate change will soon contribute to 250,000 to 4.6 million excess deaths per year based on various estimates [2–4].

A growing body of research has noted the substantial carbon emissions associated with travel for academic conferences and other educational activities [5–7]. Most of these studies have focused on the carbon costs of air travel. Other scholars, especially in the environmental sciences, have gone a step further by weighing academic objectives against travel-related carbon emissions, recognizing the need to balance knowledge building and research collaboration against carbon costs [8, 9].

The Consortium of Universities for Global Health (CUGH) is the largest organization of academic institutions in North America focusing on global health. In recent years, CUGH has recognized the growing danger of health-related effects of climate change and served as a platform for planetary health dialogue [10]. However, CUGH’s culminant conference each year since the organization’s inception in 2008 has in itself contributed to carbon emissions, particularly through participants’ air travel. Although the objectives of the conference aim to improve human health, we also must consider the substantial environmental—and therefore health and social —costs of conference-related carbon emissions.

In 2021, CUGH transitioned its annual conference (previously scheduled to take place in-person in Houston, Texas, USA) to a virtual format, because of safety concerns associated with public gatherings during the COVID-19 pandemic. Conference organizers expressed concern that this virtual format would limit ability to ‘network’, share scholarship, and develop new initiatives that were commonplace with previous ‘in-person’ annual meetings. While these potential liabilities were noted, less attention was placed on the potential benefits of the new virtual format. One of these advantages is the reduction in carbon emissions arising from the need not to travel to Houston.

The purpose of this paper is to outline an approach to quantifying the magnitude of this climate benefit so that such gains can be better appreciated in decisions regarding future in-person conferences.

## Methods

From the 2021 conference registration dataset made available from CUGH, we included each registrant who provided a home or institutional address (*n* = 1909 after excluding 40 registrants). We then calculated the distance from each individual’s place of residence to Houston, Texas. For those residing in the U.S., their home ZIP code was used. We assumed the threshold between a registrant flying versus driving was 300 miles (a commonly used distance threshold in similar travel emissions analyses) [11]. An embedded formula for Google Maps driving directions was utilized to determine driving distance [12].

For the registrants flying, we entered each person’s home city into Google Flights using April 1–3, 2022 (the date of the future 2022 CUGH conference) as departure/return dates and selected the suggested local airport with the fewest layovers and most available flights to George Bush Intercontinental Airport (IAH) in Houston, Texas. We then used the Carbon Footprint Calculator for Individuals and Households to determine the carbon footprint of individual round trip flights [13]. The Carbon Footprint Calculator is an independently audited online tool following the greenhouse gas reporting methodology outlined by the UK government [14].

For the registrants driving, we doubled the previously calculated ZIP code-based distances to Houston to estimate the round trip driving distance. We determined the total carbon footprint from each individual’s round trip drive using the average efficiency value of an average petrol car in the average van/motorbike/car database available from the Carbon Footprint Calculator website [13].

## Results

We assumed 1755 of the 1909 attendees included in the calculations would have utilized air travel, for an aggregate total of 10,675,070 round trip miles flown and 2436.14 metric tons of CO_2_ (MtCO_2_) averted. The distance traveled by those attendees utilizing air travel was bimodal, with the majority flying either 4000 miles or less or over 10,000 miles round trip (Table 1). We assumed 154 attendees would have traveled by vehicle, for an aggregate total of 28,575.2 round trip miles driven and 7.68 MtCO_2_ averted (Table 2). Altogether, we estimated the total averted carbon emissions for registrants flying and driving to and from Houston to be 2443.82 MtCO_2_ (Table 3).

**Table 1:** Number of attendees utilizing air travel

Miles per round trip	Number of attendees	Emissions averted (MtCO_**2**_)
≤ 1000	57	8.94
1001–2000	291	110.21
2001–3000	639	360.04
3001–4000	306	232.52
4001–5000	17	17.85
5001–6000	5	5.85
6001–7000	7	10.02
7001–8000	14	24.16
8001–9000	0	0
9001–10,000	30	68.46
> 10,000	389	1598.23

**Table 2:** Number of attendees traveling by vehicle

Miles per round trip	Number of attendees	Emissions averted (MtCO_**2**_)
≤ 100	74	0.12
101–300	32	1.61
301–600	48	6.07

**Table 3:** Estimated travel-related carbon emissions averted

Travel component	Carbon emissions averted (MtCO_**2**_)
Flights to/from IAH	2436.14
Driving to/from Houston	7.68
**Total**	**2443.82**

## Discussion

Based on the estimates we present here of carbon emissions associated with flying and driving to and from Houston, we determined that converting the 2021 CUGH conference from an in-person to a virtual format averted 2443.82 MtCO_2_ emissions. Moreover, these estimates are conservative in that they did not include additional sources of emissions, such as travel to and from airports, accommodation and food, and single-use paper brochures, which would have contributed to even greater carbon costs. To put these findings in perspective, 2443.82 MtCO_2_ is the emissions equivalent of 13.5 railcars of coal burned, 294 US homes’ energy use for one year, or 274,988 gal of gasoline consumed. To offset those emissions, one would need to conserve 2994 acres of U.S. forest for a year, grow 40,409 tree seedlings for a decade, or switch 92,623 incandescent bulbs to LEDs [15].

Academic sectors other than health have questioned the benefits of in-person meetings because of the very large carbon cost of travel. Even before the COVID-19 era, when many meetings transitioned to virtual formats, some academics in the ecological sciences suggested embracing virtual conferencing for environmental reasons [16]. Since the effects of climate change extend beyond environmental impacts and directly affect human health through such phenomena as rising sea levels, unbearable temperatures, and catastrophic weather events, especially in low-income and middle-income countries with vulnerable health infrastructure, it is important to consider how virtual versus in-person conferencing reflects the goals of medicine and global health [3, 17]. The differences in climate change-related helath impacts are also relevant when examining the relative per capita carbon emissions of high-income versus lower-income countries. For example, in 2018 the US emitted 13.2 MtCO_2_ per capita, while the least developed countries (by UN classification) averaged 0.335 MtCO2 per capita [18]. These data and considerations are particularly germane for CUGH since the mission is “to improve the wellbeing of people and the planet through education, research, service, and advocacy” [19].

There are, of course, valuable and intangible aspects of in-person conferencing that cannot be recreated in a virtual format, such as networking and meaningful intercultural interactions. These benefits have been examined by other global health-focused scholars [20, 21]. They can be essential to forming impactful partnerships between the Global North and Global South. There are aslo quantifiable benefits to in-person meetings, such as the formation of collaborations that result in increased research productivity [9].

Virtual conferencing has the advantage of accessibility and equity. CUGH conferences have previously been held in the US, which poses financial barriers to participants from low- and middle-income countries. Virtual conferencing eliminates those barriers.

The COVID-19 pandemic has triggered a rapid shift to online fora, and in consequence virtual meeting software has improved and become more easily accessible worldwide. Many have found that virtual meetings are valuable tools for education and dialogue in the COVID-19 era and are considering holding online conferences indefinitely [22–24]. Similarly, CUGH could consider offering non-travel-based solutions that capture some of the usual benefits of face-to-face meetings, such as designing sophisticated virtual content sessions with the secondary goals of intercultural exchange and networking in mind [25].

Moreover, the ready availability today of open-source carbon calculation formulas has made it possible for organizations to quantify each registrant’s travel-related carbon footprint and the aggregate the emissions of all conference registrants and provides a basis for transparent environmental accounting [26]. The concept of environmental accounting as a form of social responsibility has already been embraced by other healthcare entities such as in an EPA-AHA MOU in 2000 and more recently in the Planetary Health Report Card for medical schools [27, 28]. Although these publications have not yet directly reduced institutions’ carbon emissions, they have increased social awareness surrounding the effects of climate change on human health and indirectly catalyzed change at an institutional level.

Because the potential consequences of in-person versus virtual conferencing are significant for attendees, CUGH could consider making available the estimated carbon emissions associated with the conference and each registrant’s travel. The ability to compare the carbon savings of virtual attendance with the collaborative benefits of in-person attendance would be valuable for participants considering the tradeoffs of each format and would provide a basis for transparent, academic environmental accounting. Moreover, as detailed in the Methods of this study, calculating these carbon emissions for publication would not be difficult: if the conference registration form asked each individual for method of travel and estimated travel distance to the conference location, one could easily estimate each individual’s travel-related carbon footprint and aggregate the emissions of all conference registrants. Making visible CUGH’s carbon emissions would further elevate the current dialogue regarding planetary health, ideally leading to more advocacy, education, and research innovation in line with CUGH’s mission.

The estimates we present here are limited by several assumptions. For example, we do not know if all attendees reflected in this analysis would, in fact, have attended the conference in person had it been held in Houston. However, the number of registrants for this conference was only marginally greater than the number who attended the last in-person CUGH conference held in 2019 (personal communication, CUGH secretariat). We also assumed that all registrants would have taken the most direct flights or driving routes available. We likely underestimated the total carbon emissions from an in-person event because we did not account for emissions from hotel, food, other ground travel, and single-use items such as paper pamphlets because these emissions were small compared to that of air travel. On the other side of the equation, we did not measure the carbon footprint associated with internet usage in support of virtual conferencing. Although, it is relatively small, this carbon footrprint is greater than zero.

## Conclusions

Due to the urgency of the climate change crisis, healthcare organizations must examine their own real, calculable impact on planetary health, not just their aspirations. Calculating the previously unquantified carbon footprint of an international in-person conference format is essential to achieving that aim, even—and especially—if the primary goal of the conference is to better human health. CUGH and other annual academic conferences are now making choices about returning to in-person conventions or evolving to hybrid or fully virtual meetings. Because the potential consequences of in-person versus virtual conferencing are significant, we propose that academic organizations that sponsor conferences, including CUGH, consider publishing the estimated carbon emissions associated with their conferences.

## Data Availability

The datasets generated and/or analysed during the current study are not publicly available due to the private and identifying personal information contained in the dataset but are available from the corresponding author on reasonable request.

## Abbreviations

CUGH: Consortium of Universities for Global Health
IAH: George Bush Intercontinental Airport
MtCO_2_: metric tons of CO_2_
